# Diagnostic and Prognostic Value of Serum Neurofilament Light Chain in Canine Spinal Cord Diseases

**DOI:** 10.3390/vetsci12100966

**Published:** 2025-10-09

**Authors:** Chaerin Kim, Taesik Yun, Yeon Chae, Hakhyun Kim, Byeong-Teck Kang

**Affiliations:** Laboratory of Veterinary Internal Medicine, College of Veterinary Medicine, Chungbuk National University, Cheongju 28644, Republic of Korea

**Keywords:** biomarker, dog, IVDD, spinal cord diseases, NfL, serum, single-molecule array

## Abstract

This study evaluated serum neurofilament light chain (NfL) as a biomarker for spinal cord diseases in dogs, including 46 healthy dogs and 76 with conditions, such as intervertebral disc herniation (IVDH), syringomyelia (SM), fibrocartilaginous embolism (FCE), and acute non-compressive nucleus pulposus extrusion (ANNPE). There was a significant difference in serum NfL levels between healthy dogs (12.55 pg/mL) and those with spinal cord diseases (91.10 pg/mL; *p* < 0.0001). The NfL level in dogs with SM (50.7 pg/mL) was significantly lower than that in dogs with IVDH (99.3 pg/mL; *p* = 0.012) and those with other diseases, including FCE and ANNPE (241.0 pg/mL; *p* = 0.002). The area under the curve for differentiating between dogs with spinal cord diseases and healthy dogs was 0.91, with an optimal NfL cutoff value of 30.31 pg/mL (sensitivity of 80.68%; specificity of 91.30%). For dogs with IVDH treated solely with medication, the serum NfL levels in the Poor and Static group (180.0 pg/mL) were significantly higher than those in the Partial and Good group (81.30 pg/mL) (*p* = 0.03). Serum NfL is a promising biomarker for neuroaxonal injury, aiding in differentiating SM from other spinal cord diseases and evaluating treatment response.

## Simple Summary

This study evaluated serum neurofilament light chain (NfL) as a biomarker in 46 healthy and 76 dogs with spinal cord diseases, including intervertebral disc herniation (IVDH), syringomyelia (SM), fibrocartilaginous embolism, and acute non-compressive nucleus pulposus extrusion. Serum NfL levels were significantly higher in dogs with spinal cord diseases (median, 91.10 pg/mL) than in healthy dogs (12.55 pg/mL; *p* < 0.0001), with an area under the curve of 0.91 for differentiation. Notably, NfL levels in dogs with SM (50.7 pg/mL) were significantly lower than in those with IVDH (99.3 pg/mL; *p* = 0.012) and other acute conditions (241.0 pg/mL; *p* = 0.002). For medically managed IVDH cases, higher NfL levels were associated with a poorer treatment response (*p* = 0.03). Serum NfL is a promising biomarker for neuroaxonal injury, aiding in diagnosis, disease differentiation, and monitoring treatment response.

## 1. Introduction

Spinal cord diseases in dogs are critical conditions that require careful management in veterinary medicine. Significant progress has been made in this area of research. The definitive diagnosis of spinal cord diseases in dogs is based on magnetic resonance imaging (MRI) and histopathological findings [1,2]. However, the existing diagnostic methods have several limitations. For example, ante-mortem diagnosis via MRI requires anesthesia and is often limited by the high cost of examination. Moreover, general practitioners often lack specialized knowledge, making it difficult to differentiate neurological symptoms from musculoskeletal disorders, such as lameness, muscle spasms, and reduced range of motion due to joint stiffness. Consequently, the absence of MRI, often due to diagnostic inaccuracies or misdiagnoses by practitioners, frequently impedes the definitive diagnosis of neurological disorders.

There is also a lack of established biomarkers for predicting the severity of clinical symptoms and the size of lesions in spinal cord disorders [3,4,5]. The previously mentioned diagnostic methods, including MRI and histopathological findings, do not always correlate with the severity of clinical symptoms [6,7]. Moreover, the absence of biomarkers with adequate differential diagnostic power for various spinal cord diseases emphasizes the need to develop specific biomarkers that can distinguish between spinal cord diseases.

Neurofilament light chain (NfL) is the main component and the most abundant subunit of neurofilaments. Furthermore, as the most soluble subunit, it is considered the most reliable subunit of neurofilaments and can be measured in bodily fluids. NfL levels in the cerebrospinal fluid (CSF) and serum increase sharply in response to neuroaxonal damage caused by inflammation, neurodegeneration, trauma, or vascular injury [8,9,10]. Previous studies on NfL in dogs with meningoencephalitis of unknown etiology (MUE) have indicated that NfL is a highly useful biomarker of neuroaxonal injury, suggesting its potential role in diagnosing and evaluating treatment outcomes [11,12,13]. Additionally, in studies on NfL and spinal cord injury (SCI) in humans, the levels of NfL were higher in the SCI group than those in the healthy group at the time of admission [14]. Furthermore, the NfL level increased over time in cases with poor outcomes and severe secondary damage, such as ischemia [8]. This demonstrates that NfL can be a valuable indicator of SCI severity and long-term prognosis of nerve damage in cases where clinical assessment is challenging [8,14].

Spinal cord diseases in dogs are often associated with neuroinflammatory processes that contribute to disease progression [15]. The acute conditions investigated in this study, such as intervertebral disc herniation (IVDH), fibrocartilaginous embolism (FCE), and acute non-compressive nucleus pulposus extrusion (ANNPE), are all forms of acute SCI. The pathophysiology of acute SCI is biphasic, involving not only the primary mechanical insult but also a destructive secondary injury cascade that evolves over hours to weeks [15]. This secondary phase is largely characterized by neuroinflammation, a complex process including ischemia, excitotoxicity, and inflammatory cell infiltration that causes progressive neuroaxonal damage and subsequent NfL release [15]. While not all canine cases of SCI are expected to progress to severe inflammatory disorders, some may eventually develop post-traumatic syringomyelia (SM), a condition that can manifest from months to decades after the initial injury [16]. Conversely, others may evolve into leukomalacia, a condition also characterized by marked neuroinflammatory responses.

Therefore, we hypothesized that NfL concentrations would be increased in dogs with spinal cord diseases. Based on this hypothesis, this study aimed to evaluate the diagnostic utility of serum NfL, its potential to predict the severity of clinical symptoms and the lesion size in dogs with spinal cord diseases, and its potential to assess responses to medical treatment.

## 2. Materials and Methods

### 2.1. Animals

In this study, the medical records of dogs diagnosed with spinal cord disorders, including IVDH, SM, FCE, and ANNPE, were retrospectively reviewed at the Veterinary Teaching Hospital of Chungbuk National University from May 2014 to March 2024. This study was approved by the Institutional Animal Care and Use Committee of the Chungbuk National University (approval number: CBNUA-1677-22-02). The study group included 46 healthy dogs, 51 dogs with IVDH, 17 dogs with SM, 5 dogs with FCE, and 3 dogs with ANNPE. The breed composition of each group was as follows: The healthy group consisted of 9 Beagles, 8 Toy or Miniature Poodles, 5 mixed-breed dogs, 4 Bichon Frises, 4 Pomeranians, 3 Spitz, 2 Labrador Retrievers, 2 Maltese, 2 Miniature Schnauzers, 2 Welsh Corgis, 1 Shiba Inu, 1 Yorkshire Terrier, 1 Miniature Pinscher, 1 Golden Retriever, and 1 German Shepherd. The IVDH group consisted of 14 Maltese, 6 Dachshunds, 5 Yorkshire Terriers, 4 Chihuahuas, 4 Pomeranians, 4 mixed-breed dogs, 3 Pekingese, 3 Shih Tzus, 2 Cocker Spaniels, 1 Bichon Frise, 1 French Bulldog, 1 Japanese Chin, 1 Miniature Pinscher, 1 Miniature Poodle, and 1 Spitz. The SM group consisted of 5 Maltese, 4 Pomeranians, 3 Toy or Miniature Poodles, 2 Shih Tzus, 2 Chihuahuas, and 1 mixed-breed dog. The FCE group consisted of 2 Maltese, 2 Shih Tzus, and 1 French Bulldog. The ANNPE group consisted of 1 Pomeranian, 1 Miniature Poodle, and 1 mixed-breed dog.

The healthy dogs were determined to be healthy based on its history, physical examination, complete blood count, serum chemistry, and radiography. Spinal cord diseases were diagnosed using neurological evaluation and MRI.

The diagnosis of IVDH, including intervertebral disc extrusion (IVDE) and intervertebral disc protrusion (IVDP), was based on the MRI findings showing extradural spinal cord compression near the intervertebral disc space. This compression was caused by extruded or protruded disc material, appearing as a hypointense mass on T1-weighted (T1W) and T2-weighted (T2W) images [17,18]. IVDP was specifically diagnosed based on midline disc herniation, partial loss of the hyperintense nucleus pulposus signal, and a single IVDH [19]. Dogs with IVDH were treated with cage rest along with medications such as gabapentin, pregabalin, tramadol, and carprofen; combination therapy using these drugs was administered as needed.

ANNPE, a subtype of IVDH, is marked by focal T2W hyperintensity in the spinal cord, an isointense T1W region, lateralized lesions over the disc spaces, reduced volume and hyperintensity of the nucleus pulposus on T2W, and minimal extradural material with little spinal cord compression [20]. Dogs with ANNPE were prescribed pentoxifylline; in cases where pain was present, analgesics such as tramadol or gabapentin were also administered.

FCE in dogs involves fibrocartilage blocking of spinal cord blood flow, which causes sudden neurological symptoms [21]. MRI shows lateralized focal T2 hyperintensity in the gray matter, a spinal cord lesion over the vertebral body, no extradural material, and a reduced T2W hyperintense nucleus pulposus signal caudal to the lesion [22]. Dogs with FCE were prescribed pentoxifylline; in cases where pain was present, analgesics such as tramadol or gabapentin were also administered.

SM involves fluid-filled cavities in the spinal cord, primarily in the cervicothoracic region, which appear as T2 hyperintensities in the gray matter [23,24]. Dogs with SM were commonly prescribed prednisolone, omeprazole, and furosemide, with gabapentin or pregabalin added as necessary.

FCE and ANNPE were grouped as other diseases as they both cause acute, severe neuroaxonal injury, which is mechanistically distinct from the chronic process seen in IVDH and SM. Due to their small sample sizes, this grouping also improved statistical power for robust comparison with other disease categories.

### 2.2. Serum Collection

Blood samples were obtained in serum or plain tubes via jugular or peripheral venipuncture at the time of diagnosis. Blood samples were centrifuged at 2000× *g* for 10 min at room temperature. Subsequently, serum samples were separated. After collection, aliquots of the serum samples were stored at −80 °C until analysis.

### 2.3. Measurement of NfL Concentration

Serum NfL concentrations were quantified using single-molecule array (Simoa; Quanterix, Billerica, MA, USA) technology. The tests were performed using a Simoa HD-1 Analyzer (Quanterix) and a human-specific NfL assay kit, which employs an anti-NfL monoclonal antibody (Uman Diagnostics, Umeå, Sweden). Canine NfL is analogous to human NfL, and previous studies have validated the use of this kit for dogs [12,13,25,26,27,28,29]. Measurements were conducted in duplicate, according to the manufacturer’s protocol. For quality assurance, two control samples (high and low concentrations) provided with the kit were tested in duplicate during each run. The assay precision was verified if the control sample concentrations were within the specified range.

### 2.4. Clinical Severity of IVDH

A grading system was used to evaluate the extent of neurological dysfunction in each dog at admission and at 7, 14, and 30 d after treatment [30,31,32,33]. The neurological status was graded using the modified Frankel score (MFS). The MFS is classified as follows: 5, apparent pain with no neurological deficits; 4, ambulatory para/tetraparesis; 3, nonambulatory para/tetraparesis; 2, para/tetraplegia and intact superficial nociception; 1, para/tetraplegia, intact deep nociception, and absent superficial nociception; 0, para/tetraplegia and absent deep nociception [34,35]. Serum NfL levels were assessed in dogs with IVDH according to the MFS and divided into three groups: 0–2, 3, and 4–5 to facilitate intergroup comparisons.

### 2.5. Treatment Response

In patients with IVDH who underwent medical treatment without surgery, the treatment response was assessed on the 30th day of medication. As the MFS grade 5 group only experienced pain and it was difficult to evaluate the degree of further symptom improvement, the treatment response was evaluated in dogs with MFS grades of 4 or below based on their response to medical therapy: Good, when the MFS increased by 2 or more points from admission or dogs were neurologically normal; Partial, when the MFS increased by 1 point from admission; Static, when there was no change in neurological status after medication compared to admission; and Poor, when the neurologic grade was worse at day 30 than the initial score, if another clinical disability that was present at admission had developed, or if the patient had died [32,34,35,36].

### 2.6. MRI Data Analyses

MRI was performed using a 0.3-Tesla unit (Airis II, Hitachi, Tokyo, Japan) or 1.5-Tesla unit (Signa Creator, GE Healthcare, Milwaukee, WI, USA). T1W (pre- and post-contrast), T2W, and fluid-attenuated inversion recovery images were acquired in the transverse, sagittal, and dorsal planes.

A commercial image viewer (OsiriX MD v10.0; Pixmeo Sarl, Geneva, Switzerland) was used to measure the SM and IVDH lesion sizes. To identify the correlation between the lesion size of SM and NfL, quantitative syrinx measurements were performed. Quantitative measurements, including the maximum syrinx height/spinal cord height ratio-transverse plane (SHRt) and maximum syrinx cross-sectional area/spinal cord cross-sectional area ratio (SCSAR) on transverse T2W images, were obtained. The SHRt ratio was determined by dividing the maximum syrinx height by the spinal cord height on the transverse images. The SCSAR was generated by dividing the maximum syrinx cross-sectional area by the spinal cord cross-sectional area on transverse images [37,38].

To identify the correlation between the lesion size of IVDH and NfL, the spinal cord compression ratio (SCCR) and the length ratio of T2 hyperintensity were calculated. The SCCR was evaluated by comparing the cross-sectional surface area of the spinal cord at the site of disc herniation with the cross-sectional surface area of the spinal cord one vertebra cranial to the herniation on transverse T2W images [7,39,40]. If intramedullary hyperintensity was present on the T2W images, the length was measured in the sagittal T2W images and divided by the length of the L2 vertebra to calculate the T2W length ratio [41,42]. In cases in which dogs exhibited significant sagittal plane curvature of the vertebral column, the length of the abnormality was determined by measuring the combined length of multiple T2 hyperintensity lines on the sagittal images to accurately represent the true length of the affected spinal cord [42].

### 2.7. Statistical Analyses

Statistical analyses were performed using the Prism 9 software (GraphPad Software, San Diego, CA, USA). A two-tailed *p*-value < 0.05 was considered statistically significant. The Shapiro–Wilk test was performed to evaluate the normality of the data distribution, and the results indicated a deviation from normality. Consequently, nonparametric methods were used for the subsequent analyses. The Mann–Whitney U test was used to compare the differences between the two groups. Specifically, comparisons were made between the healthy and spinal cord disease groups, as well as between the two treatment response groups: Poor and Static versus Partial and Good. The Kruskal–Wallis test, followed by Dunn’s multiple comparison post hoc test, was employed to compare NfL concentrations among different spinal cord disease groups and within the IVDH group based on the MFS and SCCR. It was used to compare the NfL levels among more than three independent groups. Receiver operating characteristic (ROC) curve analysis was used to assess the diagnostic utility of NfL in differentiating healthy dogs and dogs with IVDH, SM, and other spinal cord diseases, including FCE and ANNPE. The area under the curve (AUC) was calculated, and diagnostic accuracy was categorized based on the AUC value: fail (0.5–0.6), poor (0.6–0.7), fair (0.7–0.8), good (0.8–0.9), and excellent (0.9–1.0) [43]. Pearson’s correlation test was used to assess the correlation between lesion size and NfL levels in the SM. Spearman’s rank test was used to assess the correlation between SCCR and NfL levels in the IVDH group.

## 3. Results

### 3.1. Study Cohorts

The demographic characteristics of the dogs, including age, body weight, and sex, are listed in Table 1. Patients in the IVDH group were significantly older than those in the healthy group (*p* < 0.05). Dogs with SM had significantly lower body weights than healthy dogs (*p* < 0.05). Sex did not differ between the five groups.

### 3.2. Comparison of Serum NfL Levels in Healthy Dogs and Dogs with Spinal Cord Diseases

**Figure 1:**
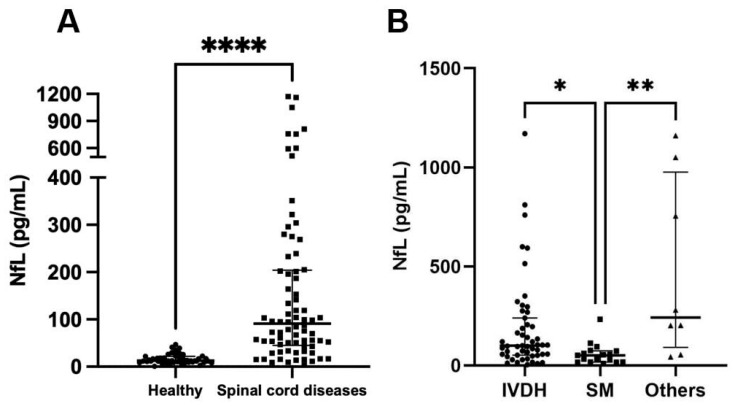
Serum NfL levels in healthy dogs and dogs with spinal cord diseases, including IVDH, SM, FCE, and ANNPE. (**A**) The serum NfL level was significantly higher in dogs with spinal cord diseases (n = 76) than that in healthy dogs (n = 46) (*p* < 0.0001) (Mann–Whitney U test). (**B**) There was a significant difference in the serum NfL level among dogs with IVDH (n = 51), SM (n = 17), and other diseases (n = 8; suspected FCE [n = 5] and ANNPE [n = 3]) (*p* = 0.001). The NfL level in dogs with SM was significantly lower than that in dogs with IVDH (*p* = 0.012) and other diseases (*p* = 0.002) (Kruskal–Wallis test with Dunn’s multiple comparison test). The horizontal bars show the medians and interquartile ranges from the first to the third quartile. * *p* < 0.05, ** *p* < 0.01; **** *p* < 0.0001. ANNPE, acute non-compressive nucleus pulposus extrusion; FCE, fibrocartilaginous embolism; IVDH, intervertebral disc herniation; NfL, neurofilament light chain; SM, syringomyelia.

Serum NfL concentrations were measured in healthy dogs and those with spinal cord diseases. In healthy dogs (n = 46), the median serum NfL level was 12.55 pg/mL (interquartile range [IQR], 9.185–21.83). In contrast, dogs with spinal cord disease (n = 76) exhibited significantly higher serum NfL levels, with a median concentration of 91.10 pg/mL (IQR, 44.88–204.3) (*p* < 0.0001; Figure 1A).

When examining specific subgroups within the spinal cord disease group, the following serum NfL concentrations were observed: dogs with IVDH (n = 51) had levels of 99.3 pg/mL (IQR, 52.17–239.4), dogs with SM (n = 17) had levels of 50.70 pg/mL (IQR, 16.62–73.9), and dogs with other diseases, such as FCE and ANNPE (n = 8), had levels of 241.0 pg/mL (IQR, 90.9–976.3). Serum NfL levels were significantly lower in dogs with SM than in dogs with IVDH (*p* = 0.012) or other diseases (*p* = 0.002, Figure 1B). However, there were no significant differences between dogs with IVDH and those with other diseases (*p* = 0.3).

### 3.3. Comparison of Serum NfL Levels in Dogs with IVDH Based on Clinical Severity

**Figure 2:**
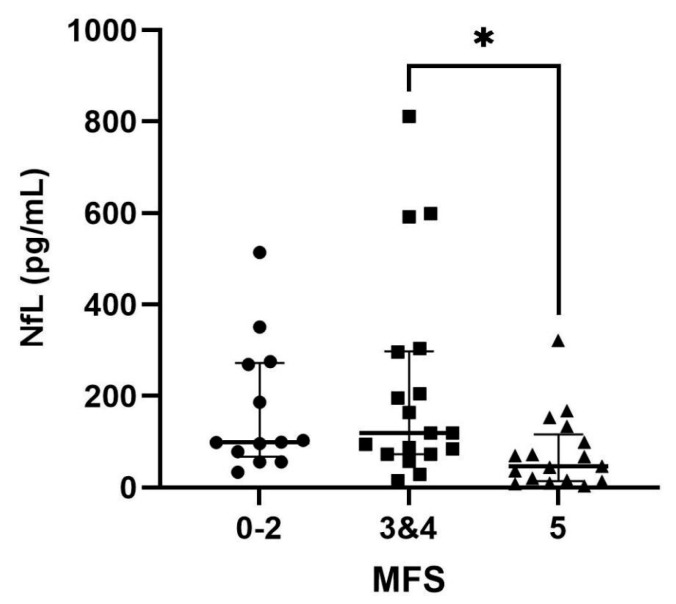
Serum NfL levels in dogs with IVDH based on the MFS. There was a significant difference in the serum NfL level among the MFS 0–2, MFS 3 & 4, and MFS 5 groups (*p* = 0.01). The NfL level of dogs with MFS 3 & 4 (n = 18) was significantly higher than that of dogs with MFS 5 (n = 17, *p* = 0.016) (Kruskal–Wallis test with Dunn’s multiple comparison test). * *p* < 0.05. IVDH, intervertebral disc herniation; MFS, modified Frankel score; NfL, neurofilament light chain.

Serum NfL levels in dogs with IVDH were analyzed based on their MFS. The NfL levels observed were 99.30 pg/mL (IQR, 67.20–272.0) for MFS 0–2 (n = 13), 119.0 pg/mL (IQR, 72.88–298.0) for MFS 3 and 4 (n = 18), and 46.90 pg/mL (IQR, 14.65–116.5) for MFS 5 (n = 17). There was a significant difference in the serum NfL levels among the different MFS groups (*p* = 0.01). The NfL levels in dogs with MFS 3 and 4 were significantly higher than those in dogs with MFS 5 (*p* = 0.016) (Figure 2). However, MFS 0–2 did not show a significant difference compared with the other two groups.

### 3.4. Serum NfL Levels in Dogs with IVDH Based on Treatment Response

**Figure 3:**
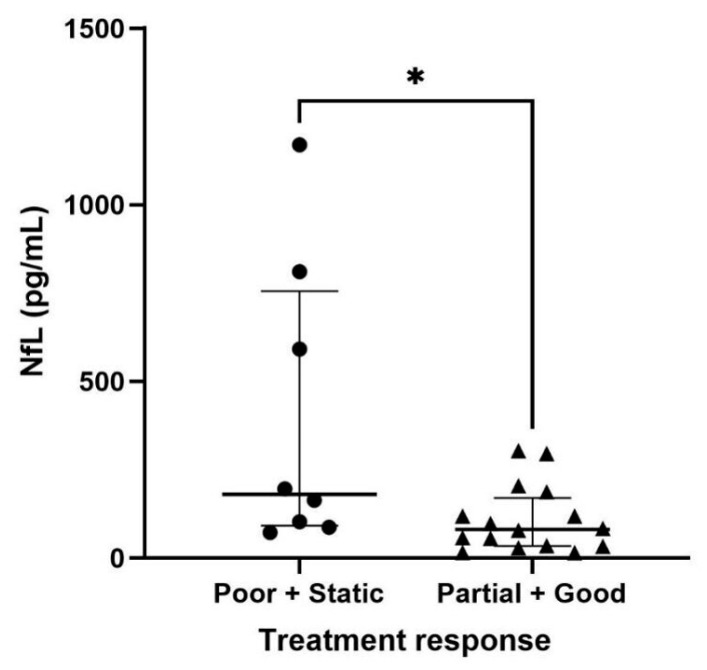
Serum NfL levels in dogs with IVDH based on treatment response. The groups were categorized as Poor & Static (n = 8) and Partial & Good (n = 16). The NfL level of dogs in the Poor & Static group was significantly higher than that of dogs in the Partial & Good group (*p* = 0.03) (Mann–Whitney U test). * *p* < 0.05.

The serum NfL levels in dogs with IVDH were analyzed based on their response to treatment. The serum NfL levels observed were 180.0 pg/mL (IQR, 91.60–756.3) for the Poor and Static group (n = 8) and 81.30 pg/mL (IQR, 34.02–170.0) for the Partial and Good groups (n = 16). There was a significant difference in serum NfL levels between the two groups (*p* = 0.03) (Figure 3).

### 3.5. ROC of NfL Concentration in Healthy Dogs and Dogs with Spinal Cord Diseases

**Figure 4:**
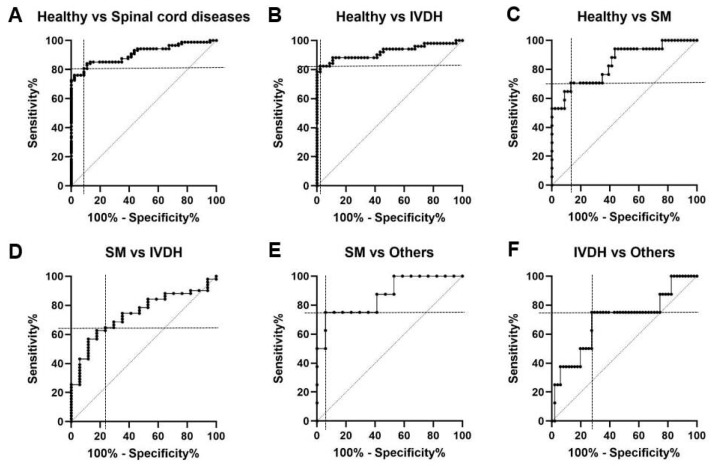
The ROC of NfL concentration in healthy dogs and dogs with spinal cord diseases. ROC curve illustrates the sensitivity and specificity of NfL to distinguish healthy dogs from those with spinal cord diseases (**A**–**C**). The areas under the curve (AUCs) are as follows: (1) healthy and spinal cord disease dogs, 0.91 (95% confidence interval [CI] = 86.22–95.80%); (2) healthy and IVDH dogs, 0.92 (95% CI = 86.17–98.05%); and (3) healthy and SM dogs, 0.84 (95% CI = 72.86–95.94%). The corresponding optimal cutoffs (sensitivity and specificity) for the NfL are as follows: (1) healthy and spinal cord disease dogs, 30.31 pg/mL (81.58% [95% CI = 71.42–88.70%] and 91.30%, [95% CI = 79.68–96.57%]), respectively; (2) healthy and IVDH dogs, 42.60 pg/mL (82.35% [95% CI = 69.75–90.43%] and 97.83% [95% CI = 88.66–99.89%]), respectively; and (3) healthy and SM dogs, 27.45 pg/mL (70.59% [95% CI = 46.87–86.72%] and 86.96% [95% CI = 74.33–93.88%]), respectively. The ROC curve illustrates the sensitivity and specificity of using NfL levels to distinguish between IVDH, SM, and other diseases, including ANNPE and FCE (**D**–**F**). The AUCs are as follows: (1) SM and IVDH dogs, 0.74 (95% CI = 61.91–86.41%); (2) SM and other disease dogs, 0.86 (95% CI = 70.86–100%); and (3) IVDH and other disease dogs, 0.69 (95% CI = 48.23–91.48%). The corresponding optimal cutoffs (sensitivity and specificity) for the NfL are as follows: (1) SM and IVDH dogs, 72.55 pg/mL (64.71% [95% CI = 50.99–76.37%] and 76.47%, [95% CI = 52.74–90.44%]), respectively; (2) SM and other disease dogs, 156.2 pg/mL (75.00% [95% CI = 40.93–95.56%] and 94.12% [95% CI = 73.02–99.70%]), respectively; and (3) IVDH and other disease dogs, 198.5 pg/mL (75.00% [95% CI = 40.93–95.56%] and 72.55% [95% CI = 59.05–82.89%]), respectively. ANNPE, acute non-compressive nucleus pulposus extrusion; FCE, fibrocartilaginous embolism; IVDH, intervertebral disc herniation; NfL, neurofilament light chain; ROC, receiver operating characteristic; SM, syringomyelia.

ROC curve analysis was used to evaluate the effectiveness of NfL in differentiating healthy dogs from those with spinal cord diseases, including IVDH, SM, ANNPE, and FCE. The analysis revealed excellent accuracy, with an AUC of 0.91 for distinguishing between healthy dogs and those with any spinal cord diseases. The optimal NfL cutoff was 30.31 pg/mL (80.68% sensitivity and 91.30% specificity). In more specific comparisons, the AUC for healthy dogs versus those with IVDH was slightly high at 0.92, with a cutoff of 42.60 pg/mL (82.35% sensitivity; 97.83% specificity). Conversely, for healthy dogs versus those with SM, the AUC was 0.84, with a lower cutoff of 27.45 pg/mL (70.59% sensitivity; 86.96% specificity) (Figure 4A–C).

ROC curve analysis was performed to differentiate the spinal cord diseases. The AUC for distinguishing dogs with SM from those with IVDH was 0.74, using a cutoff of 72.55 pg/mL (64.71% sensitivity; 76.47% specificity). The AUC for differentiating dogs with SM from those with other diseases was 0.86, with a cutoff of 156.2 pg/mL (75% sensitivity, 94.12% specificity). The AUC for differentiating dogs with IVDH from those with other diseases was 0.69, with a cutoff of 198.5 pg/mL (75% sensitivity and 72.55% specificity) (Figure 4D–F).

### 3.6. Correlation Between the Serum NfL Level and the Lesion Size in Dogs with SM

**Figure 5:**
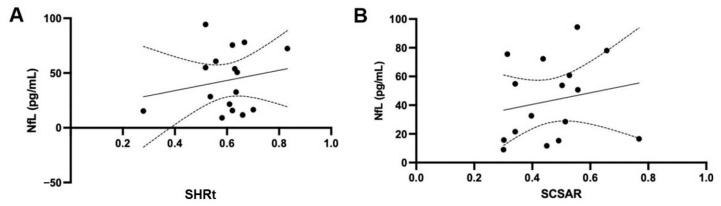
Correlation between the serum NfL level and the lesion size in dogs with SM. The serum NfL level in dogs with SM showed no significant correlation with SHRt (n = 16, r = 0.193, *p* = 0.47) (**A**) and SCSAR (n = 16, r = 0.194, *p* = 0.47) (**B**). Dotted lines represent the 95% confidence intervals. (Pearson’s correlation test). NfL, neurofilament light chain; SCSAR, maximum syrinx cross-sectional area/spinal cord cross-sectional area ratio on transverse images; SHRt, maximum syrinx height/spinal cord height ratio on transverse images; SM, syringomyelia.

This study investigated the correlation between NfL concentration and the size of syrinx lesions in dogs using two quantitative measurements on transverse MRI images. Pearson’s correlation test showed no significant correlation for either SHRt (r = 0.193, *p* = 0.47, n = 16; Figure 5A) or SCSAR (r = 0.194, *p* = 0.47, n = 16; Figure 5B).

### 3.7. Correlation of Serum NfL Levels with the Lesion Size in Dogs with IVDH

**Figure 6:**
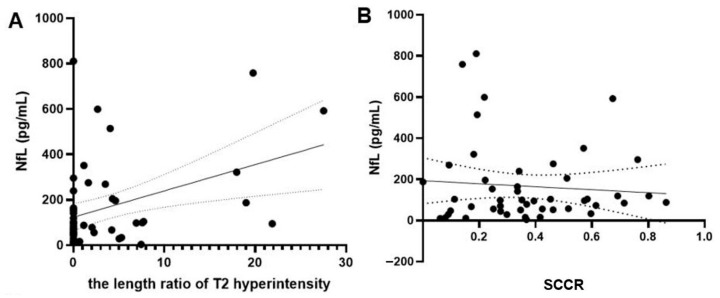
Correlation of serum NfL levels with the lesion size in dogs with IVDH. Correlation between the serum NfL level and the length ratio of T2 hyperintensity in dogs with IVDH was significant (n = 48, r = 0.290, *p* = 0.046) (**A**). Conversely, there is no significant correlation between the serum NfL level and the SCCR in dogs with IVDH (n = 48, r = 0.068, *p* = 0.65) (**B**). Dotted lines represent the 95% confidence intervals (Spearman’s rank test). SCCR, spinal cord compression ratio.

There was a mildly positive correlation between serum NfL levels and the T2 hyperintensity length ratio in dogs with IVDH, with Spearman’s correlation coefficient (r) of 0.290 and *p* = 0.046 (n = 48) (Figure 6A). In contrast, the correlation between serum NfL levels and the IVD compression ratio in dogs with IVDH was not significant, as indicated by Spearman’s correlation coefficient (r) of 0.068 and a *p*-value of 0.65 (n = 48) (Figure 6B).

## 4. Discussion

In this study, serum NfL levels allowed for a comparison between healthy dogs and those with spinal cord diseases. First, the evaluation of NfL levels facilitated the differentiation of SM from other spinal cord diseases. Second, NfL concentrations in IVDH correlated with clinical symptoms in dogs with mild to moderate disease severity. Third, the response of dogs with moderate-to-severe clinical signs of IVDH undergoing medical treatment was predicted. Therefore, serum NfL concentration could be a potential biomarker for identifying spinal cord diseases and differentiating SM from other spinal cord diseases. This could also be beneficial in predicting secondary injuries caused by IVDH.

The diagnosis of spinal cord diseases in dogs has traditionally been conducted primarily through neurological examinations and MRI, with definitive diagnoses for certain conditions, such as neoplasia and immune-mediated diseases, often confirmed postmortem via histopathological examinations [44]. MRI, the most commonly used imaging modality, requires anesthesia, which limits its use in dogs with systemic diseases. Although access to MRI has recently improved, it remains a high-cost procedure. To overcome these limitations, researchers have explored the use of affordable and easily measurable biomarkers, such as C-reactive protein, creatine kinase, and lactate, for the diagnosis and prognosis of dogs with IVDH [45,46,47]. However, these biomarkers are nonspecific and do not adequately reflect neural damage. A novel biomarker, NfL, has emerged to overcome the limitations of the aforementioned biomarkers in human spinal cord diseases. NfL concentration sharply rises in response to neuroaxonal injury, with its concentration in the blood being approximately 40 times lower than that in the CSF [9]. Considering the comprehensive characteristics of NfL, it fulfils the criteria for an ideal biomarker, including the ability to be easily measured in collected samples, possessing sensitivity and specificity for the disease, and allowing for repeated measurements [48]. Therefore, this study investigated the utility of NfL as a biomarker of spinal cord diseases in dogs.

In studies that have evaluated the diagnostic accuracy of various biomarkers in human SCI, both CSF and serum NfL have emerged as effective screening biomarkers [49]. In SCI, NfL levels increase because of both primary and secondary injury mechanisms. Primary injury involves immediate mechanical damage, which releases NfL from the axons. Secondary injury processes, including ischemia, inflammation, oxidative stress, and excitotoxicity, further damage neurons and axons, resulting in additional NfL release [15]. In this study, dogs with spinal cord diseases had higher serum NfL concentrations than healthy dogs. Moreover, similar to human studies, the ability to differentiate dogs with spinal cord diseases from healthy dogs showed excellent diagnostic accuracy. Neurological symptoms are often difficult to differentiate because of limited information and the vagueness of symptoms. Lameness is usually a sign of musculoskeletal disorders but can occasionally result from neurological conditions, such as root signs [44]. Additionally, for general practitioners, the limitations of specialized knowledge make it difficult to distinguish neurological symptoms, leading to frequent misdiagnoses. Comparing dogs with spinal cord diseases with healthy dogs revealed an increase in serum NfL levels in the former, indicating that serum NfL could be a useful screening tool for spinal cord diseases in dogs.

The serum NfL concentration in dogs with SM was significantly lower than that in dogs with IVDH, FCE, and ANNPE. This study demonstrated the diagnostic utility of distinguishing dogs with SM from those with IVDH and other diseases, including ANNPE and FCE, showing high diagnostic accuracy. The difference in NfL concentrations between SM and other spinal cord diseases can be attributed to their distinct pathophysiologies and the nature of onset. ANNPE and FCE exhibit acute symptoms due to sudden trauma and vascular blockage, respectively [18,20,50]. Similarly, most IVDH cases in this study were classified as IVDE, which led to acute clinical signs. Among the 51 patients with IVDH, 27 presented with either extrusion alone or a combination of extrusion and protrusion. IVDE results from sudden nucleus pulposus extrusion into the spinal canal, causing severe SCI through compressive and contusive forces [51,52,53]. This acute condition, caused by ANNPE, FCE, and IVDE, results in significant axonal damage, a glial response dominated by phagocytic microglia/macrophages, and a proinflammatory environment similar to that of human SCI, leading to severe neurological deficits [54]. In contrast, SM, often secondary to Chiari-like malformations, is a chronic condition characterized by symptoms that develop gradually over weeks, months, or years [23]. SM involves abnormal CSF dynamics, leading to fluid accumulation in the spinal cord and neuroaxonal injury [55]. Inflammatory responses, including macrophage activation, exacerbate tissue damage and neurodegeneration. Additionally, the expanded perivascular spaces disrupt blood flow and nutrient delivery, thereby contributing to additional neuroaxonal damage [16]. As SM causes slower and less severe neural damage than other conditions, these pathophysiological differences lead to varying serum NfL levels, thereby supporting the differentiation of SM from other spinal cord diseases. Therefore, measuring serum NfL levels could aid in distinguishing SM from other conditions, such as IVDE, ANNPE, and FCE, when combined with clinical symptoms.

In dogs with IVDH, serum phosphorylated neurofilament-heavy chain elevation can predict outcomes of progressive myelomalacia secondary to Type I IVDH [56]. Therefore, we examined whether NfL levels could predict the severity of clinical symptoms or prognosis in dogs with IVDH. Serum NfL levels in the MFS grades 3 and 4 group, which had more severe clinical symptoms, were significantly higher than those in the MFS grade 5 group. However, serum NfL levels in the MFS grade 0–2 group, despite having more severe symptoms, were not significantly higher than those in the MFS grades 3 and 4 group. This suggests that NfL levels are associated with clinical symptoms in dogs with mild to moderate clinical IVDH severity. Previous studies have observed that pathological changes in the spinal cord due to IVDE vary significantly, from undetectable alterations to severe hemorrhage and necrosis, suggesting that clinical symptoms may not always align with histopathological findings [57]. Certain clinical signs, such as symptom duration, Schiff–Sherrington posture, loss of reflexes, and pain on spinal palpation, do not necessarily reflect the histopathological severity of spinal cord damage. In this study, moderate clinical symptoms (MFS 3–4) were suspected to be associated with active neurodegeneration, resulting in significantly higher NfL concentrations than those in mild clinical signs (MFS 5). However, in severe cases (MFS 0–2), while NfL levels may be elevated owing to extensive neuroaxonal injury, the increase may not be as pronounced because of the substantial loss of neurons, which are the primary source of NfL. Therefore, severe symptoms could result in greater variations in NfL levels than those caused by mild to moderate symptoms.

Current research on the prognosis of IVDH in dogs has predominantly focused on surgical cases [58]. This is because the loss of deep pain in dogs indicates the need for surgery and regaining the ability to walk after surgery is crucial [2,58]. However, a significant number of patients were treated with medication alone, highlighting the need to evaluate the improvement in clinical symptoms in medication-only subjects. There was a significant difference in serum NfL levels between the Poor and Static and the Partial and Good groups, with the latter group showing lower NfL levels. Previous studies in humans have shown that NfL is an effective biomarker for monitoring treatment responses in neurological disorders, such as multiple sclerosis and neuromyelitis optica spectrum disorder. Elevated NfL levels are associated with great axonal damage and poor treatment responses [59,60]. In veterinary medicine, NfL concentration is also a useful biomarker for assessing treatment responses in dogs with MUE [12,13]. Although definitive conclusions could not be drawn without including all groups, elevated NfL levels at presentation may reflect the extent of spinal cord damage, suggesting that high NfL levels could be a negative prognostic indicator of response to medication.

In the present study, there was no correlation between serum NfL levels and lesion size in dogs with SM. Studies on the relationship between spinal cord lesions and NfL concentrations in humans have confirmed that elevated NfL levels are associated with the degree of inflammation. Different underlying mechanisms in diseases that affect the spinal cord, such as neuromyelitis optica spectrum disorders and spinal cord infarctions, show higher NfL levels due to more severe axonal injury caused by intense inflammatory processes or ischemic necrosis [61,62]. Because SM in humans is a chronic disease with generally mild inflammation, unless lesions are significantly enlarged, the degree of inflammation is less noticeable relative to lesion size compared to other diseases [63]. Consequently, this difference in inflammation severity resulted in a weak correlation between lesion size and NfL concentration in our study.

In the MRI evaluation of IVDH in dogs, understanding the significance of T2W-signal intensity and SCCR, as well as their relationship with neuronal injury, is crucial. T2W hyperintensity, caused by secondary damage, such as hemorrhage, malacia, edema, and necrosis, directly reflects the severity of injury within the spinal cord tissue [64,65,66]. Consequently, a longer T2 hyperintensity observed with low-field MRI (0.3T and 1T) is associated with more severe secondary injury and may indicate a worse prognosis [17,34,35]. However, recent studies using high-field MRI (3T) have shown a poor correlation between T2W hyperintensity and prognosis, likely owing to an improved signal-to-noise ratio and high image resolution. With high-resolution imaging, distinguishing significant pathological changes from less significant abnormalities becomes more challenging, thus reducing the specificity of T2W hyperintensity as a prognostic marker [67,68].

In contrast, SCCR measures the degree of external mechanical compression but does not directly indicate neuronal injury. This implies that similar compression levels can result in varying degrees of SCI depending on the concussive forces involved during the extrusion event [7]. Consequently, there was no significant correlation between the intervertebral compression ratio and prognosis in dogs.

This study employed MRI to explore the potential positive correlation between T2W hyperintensity and NfL levels in dogs, based on the assumption that T2W hyperintensity correlates with neuronal injury. A mildly positive correlation between serum NfL levels and T2 hyperintensity length ratio was found in dogs with IVDH, suggesting that NfL levels can quantitatively reflect structural changes and SCI. However, there was no correlation between serum NfL levels and SCCR in these dogs. These results imply that, while T2W hyperintensity is a direct indicator of neuronal injury, SCCR is less effective in reflecting this injury. Consequently, differences in how T2W hyperintensity and SCCR are related to neuronal injury lead to variations in their correlations with NfL levels. More severe disc herniation increases the likelihood of injury but does not necessarily reflect the extent of damage, indicating that other factors may critically influence the overall prognosis.

Vasogenic edema is a key component of the neuroinflammatory cascade that drives secondary injury in SCI [15], and as noted, is a target for effective anti-inflammatory agents in humans. In clinical veterinary practice, the T2 hyperintensity we measured serves as the most direct MRI correlate for parenchymal edema and hemorrhage [64], as well as other pathologies like necrosis [54]. The significant positive correlation we found between the T2 hyperintensity length ratio and serum NfL levels (*p* = 0.046) strongly supports this link. This finding suggests that serum NfL is not just a marker of primary axonal injury, but also quantitatively reflects the severity of secondary neuroinflammatory processes, including vasogenic edema. We agree that a more specific characterization of vasogenic edema in the canine spinal cord using advanced imaging techniques is a critical next step for future research.

This study had several limitations. First, the sample size of dogs with ANNPE and FCE was not large enough to represent a broader cohort. Second, only the serum NfL levels were measured. Given that NfL is released from neurons into the CSF and subsequently into the peripheral blood when axonal damage occurs, serum samples may not reflect the extent of neural damage as accurately as the CSF. Therefore, future studies should compare serum and CSF NfL levels. Third, because NfL levels were analyzed at 30 d to evaluate treatment response, long-term prognosis was not assessed in this study. Fourth, this was a retrospective study, and as such, we did not systematically evaluate MRI signal changes, such as T2 hyperintensity indicative of vasogenic edema, around the syrinx in the SM group. The presence of significant peri-syrinx edema could suggest ongoing active inflammation, which may have influenced serum NfL concentrations in some individuals. Future prospective studies should include a quantitative analysis of these peri-lesional MRI changes to better characterize the inflammatory status of SM and its correlation with NfL levels.

## 5. Conclusions

This study demonstrated that NfL is a highly promising biomarker for neuroaxonal injury in canine spinal cord diseases. This could serve as a screening tool to differentiate between SM and other spinal cord diseases in patients presenting with clinical signs, such as pain, ataxia, and paralysis. Serum NfL has the potential to identify structural spinal cord diseases in dogs, thereby supporting the use of advanced diagnostic procedures, such as MRI. In the case of IVDH, NfL levels can quantitatively reflect SCI, making them useful for assessing the degree of neuronal injury at diagnosis, especially in cases where anesthesia is challenging, and for monitoring the progression of injury over time in diagnosed patients. In addition, NfL levels can aid in estimating the prognosis of medical treatment responses.

## Figures and Tables

**Table 1:** Demographics of dogs included in this study.

	Healthy	IVDH	SM	FCE	ANNPE
	(n = 46)	(n = 51)	(n = 17)	(n = 5)	(n = 3)
Age (years)	2 (1–3)	9 ^a^ (6–12)	5 (3–7)	8 (4–8)	3 (2–5)
Body weight (kg)	6.38 ^b^ (4.48–8.72)	5.2 (3.6–6.85)	3.56 (3.04–4.62)	5.83 (5.6–6.5)	6.7 (6.1–7.03)
Sex (number)					
Male	19 (41%)	30 (59%)	10 (59%)	2 (40%)	2 (66%)
Female	27 (59%)	21 (41%)	7 (41%)	3 (60%)	1 (33%)

The results are expressed as medians and interquartile ranges. The values for sex are expressed as the number of dogs (percentage of total instances). This value is significantly higher compared to ^a^ Healthy and ^b^ SM (*p* < 0.05). Differences among the five groups are evaluated using the Kruskal–Wallis test for age and body weight, and the chi-square or Fisher’s exact test for sex. ANNPE, acute non-compressive nucleus pulposus extrusion; FCE, fibrocartilaginous embolism; IVDH, intervertebral disc herniation; SM, syringomyelia.

## Data Availability

The original contributions presented in this study are included in the article. Further inquiries can be directed to the corresponding author.
